# The Antidepressant Sertraline Induces the Formation of Supersized Lipid Droplets in the Human Pathogen *Cryptococcus neoformans*

**DOI:** 10.3390/jof8060642

**Published:** 2022-06-17

**Authors:** Matthew R. Breuer, Ananya Dasgupta, Joseph G. Vasselli, Xiaorong Lin, Brian D. Shaw, Matthew S. Sachs

**Affiliations:** 1 Department of Biology, Texas A&M University, College Station, TX 77843, USA; mbreuer@bio.tamu.edu (M.R.B.); adasgupta@bio.tamu.edu (A.D.); 2 Department of Plant Pathology and Microbiology, Texas A&M University, College Station, TX 77843, USA; joe723@tamu.edu (J.G.V.); brian.shaw@ag.tamu.edu (B.D.S.); 3 Department of Microbiology, University of Georgia, Athens, GA 30602, USA; xiaorong.lin@uga.edu

**Keywords:** antifungal therapy, *Cryptococcus neoformans*, *Aspergillus fumigatus*, *Candida albicans*, *Saccharomyces cerevisiae*, lipid droplets

## Abstract

The prevalence and increasing incidence of fungal infections globally is a significant worldwide health problem. Cryptococcosis, primarily caused by the pathogenic yeast *Cryptococcus neoformans*, is responsible for approximately 181,000 estimated deaths annually. The scarcity of treatments and the increasing resistance to current therapeutics highlight the need for the development of antifungal agents which have novel mechanisms of action and are suitable for clinical use. Repurposing existing FDA-approved compounds as antimycotic therapeutics is a promising strategy for the rapid development of such new treatments. Sertraline (SRT), a commonly prescribed antidepressant, is a broad-spectrum antifungal agent with particular efficacy against *C. neoformans*. However, the effect of SRT on fungal physiology is not understood. Here, we report that SRT induces the formation of supersized lipid droplets (SLDs) in *C. neoformans*, and in *Candida albicans*, *Saccharomyces cerevisiae*, and *Aspergillus fumigatus*. SLDs were not induced in *C. neoformans* by treatment with the antifungal fluconazole (FLC), consistent with SRT and FLC acting differently to perturb *C. neoformans* physiology. The formation of SLDs in response to SRT indicates that this compound alters the lipid metabolism of *C. neoformans*. Moreover, the SRT-induced enlargement of LDs in other fungal species may indicate a common fungal response to SRT.

## 1. Introduction

A billion fungal infections occur annually. More than 150 million of these are considered severe and result in 1.6 million deaths each year [1]. Therefore, fungal infections remain a serious threat to global human health [2]. One fungal pathogen, *Cryptococcus neoformans,* is a ubiquitous basidiomycetous yeast associated with diverse ecological niches [3]. Infection with *C. neoformans* generally occurs by inhaling airborne yeasts or basidiospores, which are then deposited into the pulmonary alveoli. The fungal cells can cause pulmonary cryptococcosis in immunocompromised hosts and disseminate to other body regions via the bloodstream. In ~90% of cases, fungi disperse to the central nervous system (CNS), resulting in cryptococcal meningitis [4]. An estimated 220,000 cases of cryptococcal meningitis occur annually and the worldwide mortality rate when contracted is 81% [5]. Current treatment relies on induction therapy with amphotericin B with or without flucytosine, followed by azole treatment [6,7].

Repurposing existing therapeutics as antimycotics is a promising approach to accelerate the antifungal development process [7,8,9]. This approach is advantageous over de novo drug discovery methods because established safety and pharmacological profiles exist for promising compounds. The selective serotonin reuptake inhibitor sertraline (SRT) is prescribed to treat mood disorders, including depression and social anxiety disorder [10]. Importantly, it also exhibits antifungal activity [11].

In humans, SRT increases serotonin levels by directly inhibiting the sodium-dependent serotonin receptor, blocking serotonin reuptake at both the dendrites and axons [10]. SRT is approved by the FDA and is safe for long-term use [12,13,14]. Previously, we screened the Johns Hopkins Clinical Compound Library and identified SRT as a broad-spectrum antifungal agent [11]. SRT exhibits particular efficacy against a wide variety of *Cryptococcus* clinical isolates at physiologically relevant concentrations in vitro (≤10 μg/mL) and demonstrates efficacy against cryptococcosis in mouse models [11,15,16]. SRT is effective as an antifungal by itself and in combination with established antifungal therapeutics, including fluconazole (FLC) and amphotericin B [11,17,18,19]. Moreover, SRT effectively penetrates the blood–brain barrier to accumulate at high concentrations in the CNS [20,21,22]. Unfortunately, the results from clinical trials using therapeutic amounts of FLC with additional SRT during maintenance therapy indicate that SRT does not increase the efficacy of treatment [23,24,25], likely due to insufficient duration of therapeutic SRT concentrations.

Nonetheless, the discovery of SRT’s target(s) in fungi could facilitate the directed design of antimycotic drugs with novel mechanisms of action. Currently, the targets through which SRT exerts its anti-cryptococcal activities remain unknown, and several hypotheses have been proposed to explain its antifungal activity. Studies on *Saccharomyces cerevisiae* suggest that SRT may insert into the phospholipid membranes of intracellular organelles, which is accompanied by eventual cell death [26,27]. There is also evidence that SRT interferes with translation [11].

Here, we report that SRT induces the formation of supersized lipid droplets (SLDs) in *C. neoformans* in vitro. Lipid droplets (LDs) are dynamic intracellular organelles that possess a unique structure. LDs consist of a neutral lipid core, primarily triacylglycerols and sterol esters, surrounded by a phospholipid monolayer (not a bilayer) [28]. The monolayer is associated with LD-specific integral and peripheral membrane proteins that participate in multiple processes, including the regulation of LD size and localization. Although LDs were long assumed to be inert fat particles, existing solely to store energy-rich compounds, recent studies have revealed that LDs participate in many essential cellular functions, including membrane trafficking, phospholipid recycling, intracellular protein metabolism, and cell signaling [28,29].

In this study, we show that *C. neoformans* cells form SLDs in response to SRT treatment and that SRT-induced SLDs form by the fusion of smaller LDs. Additionally, we show that SRT induces the formation of SLDs in the model yeast *S. cerevisiae*, the pathogenic yeast *Candida albicans*, and the pathogenic filamentous fungus *Aspergillus fumigatus*, signifying that the effect of SRT on fungal LDs is not specific to *Cryptococcus*. While SRT has been proposed to be cytotoxic to *S. cerevisiae* through non-specific interactions with phospholipids, no evidence for SLD formation in response to SRT has been reported in fungi [26,27]. The formation of SLDs in response to SRT could thus be important to SRT’s mechanism of antifungal activity.

## 2. Materials and Methods

### 2.1. Strains, Media, and Drug Treatments

The fungal strains used in this study were *C. neoformans* strain H99 and LK62 (Cdc10-mCherry), *S. cerevisiae* strain S288c, *C. albicans* strain SC5314, and *Aspergillus fumigatus* strain Af293. Strains were maintained as −80 °C glycerol stocks and streaked onto YPD solid medium prior to use. For the experiments, *C. neoformans* overnight cultures were started from a single colony and were grown at 37 °C, 150 RPM for 12–18 h. The medium used was RPMI-1640 liquid medium (Sigma, Cat. R1383, Sigma-Aldrich, St. Louis, MO, USA) supplemented with 2% glucose and 165 mM MOPS, adjusted to pH 7 with NaOH (this glucose-supplemented RPMI-1640 medium is referred to as RPMI-1640 throughout this communication). *S. cerevisiae* and *C. albicans* strains were grown similarly.

Overnight cultures were used to inoculate fresh RPMI cultures for drug treatments as described in the figure legends. Drug stocks were stored at −20 °C. Sertraline (Matrix Scientific, Cat. 047897, Matrix Scientific, Columbia, SC, USA) stocks were prepared by dissolving powder to 20 mg/mL in DMSO, with subsequent dilution to 2 mg/mL with sterile MilliQ water. Fluconazole (Alfa Aesar, Cat. J62015, Thermo Fisher Scientific, Waltham, MA, USA) stocks were prepared by dissolving powder to 2 mg/mL in DMSO, with subsequent dilution to 0.2 mg/mL with sterile MilliQ water. Prior to dilution with water, drug stocks in DMSO were filter-sterilized using DMSO-compatible Corning 0.2 μm filters (Corning, Cat 431222, Corning Inc., Corning, NY, USA). In cases where cells were fixed prior to imaging, fixation was accomplished by resuspension of cell pellets in 4% paraformaldehyde and incubation on the benchtop for 15 min. Additional details are given in the figure legends.

The MIC_50_ (minimum inhibitory concentration required to inhibit 50% of the growth of organisms) was measured as follows. H99 cells were inoculated at a concentration of 10^5^ cells/mL in RPMI-1640 media at 37 °C with 150 rpm shaking and cultured with 1% DMSO (vehicle control) or in the presence of increasing concentrations of SRT, FLC, or SRT and FLC. Growth studies were performed in triplicate. After 12 h, aliquots of cell suspensions were serially diluted and plated on YPD agar and incubated at 30 °C for two days to assess cell viability by colony formation.

### 2.2. LD Staining

BODIPY 493/503 (Cayman Chemical, Cat. 25892, Cayman Chemical, Ann Arbor, MI, USA) stock was prepared by dissolving powder to 3.8 mM in DMSO. To stain LDs, a 10 μM (2X) staining solution was prepared in fresh RPMI-1640 medium (live-cell staining) or in PBS (fixed-cell staining). In total, 1 mL of cells in an Eppendorf tube was pelleted by centrifugation (5000× *g* for 4 min), and 500 μL supernatant was removed by pipetting. The 2X staining solution was vortexed vigorously for ~15 s as recommended by the supplier, and then 500 μL of staining solution was immediately added to the cell pellet and remaining supernatant, and the cells were resuspended by gentle vortexing. Samples were incubated for 30 min in a ThermoMixer (25 °C, 750 RPM) covered with foil to protect from light. Cells were then washed three times with PBS by pelleting and resuspension in PBS before being resuspended in fresh RPMI-1640 medium or PBS. Cells were then prepared for imaging as described below.

MDH (Abcepta, Cat. SM1000a, Abcepta, Inc., San Diego, CA, USA) was also used to stain LDs in live cells. A 2X staining solution (0.2 μM MDH) was freshly prepared by the dilution of the stock in RPMI-1640 medium. In total, 1 mL of cells was centrifuged (4500× *g* for 5 min), and 500 μL supernatant was aspirated away. The 2X staining solution was vortexed vigorously for ~15 s, then 500 μL was immediately added to the cell pellet and remaining supernatant. Cells were resuspended by gently pipetting up and down. Samples were incubated for 15 min in a ThermoMixer (25 °C, 750 RPM) covered with foil and then washed and prepared for imaging as described for the BODIPY493/503 staining.

### 2.3. Imaging and Post-Acquisition Processing

Live cells were imaged within a chamber modified from a design previously described by Hoch [30]. The chamber was produced by 3D printing using polyvinylpyrrolidone (Supplementary File S1). Briefly, a poly-d-lysine-coated coverslip (22 × 22 mm, #1.5) was adhered to the bottom of the chamber using vacuum grease. Small volumes of samples (7–10 μL) were loaded into the chamber (typically, four samples were loaded). The samples were incubated on the benchtop for 2–5 min to allow the cells to settle to the coverslip. The chamber was sealed by attaching an additional coverslip to the top of the chamber with vacuum grease. Alternatively, if fixed cells were to be imaged, 3–5 μL of fixed cell sample was loaded onto a poly-d-lysine-coated coverslip and mounted onto a microscope slide. Both live and fixed samples were imaged using an Olympus FV3000 confocal microscope equipped with a 100× TIRF objective (NA 1.49). Images were acquired using the native FluoView software (Olympus, Center Valley, PA, USA), and raw image files were imported into FIJI for processing (ImageJ v2.1.0/1.53c).

To visualize LDs by fluorescence microscopy, the lipid stains BODIPY 493/503 and MDH were excited using the 488 nm and 405 nm laser lines, respectively. We also acquired images of the transmitted-light channel. We imaged every 0.32 µm on average with a total depth of 6–10 μm. This yielded Z-stacks consisting of ~15–30 frames. For the processing of multi-channel hyperstacks, the raw image files were imported into FIJI and separated into single-channel Z-stacks. Stacks from the transmitted channel were Z-projected by minimum intensity, while stacks from the fluorescence channels were Z-projected by maximum intensity. Measurements of LD sizes were conducted in FIJI; the native line tool was used to measure the LD diameters. The resulting measurements were exported and plotted.

Time-lapse movies of live cells in the transmitted channel of both DMSO- and SRT-treated cells were acquired in the same session using multi-area time-lapse imaging (MATL), which facilitates the imaging of multiple defined XY coordinates in series. Auto-focusing to the same Z-plane was facilitated by automated z-drift compensation throughout the experiment. Images of the same fields of view were acquired at a rate of 1 image/minute, from 96–348 min, following the additions of DMSO and SRT. Movies were generated from the time-lapse datasets in FIJI.

## 3. Results

**Figure 1:**
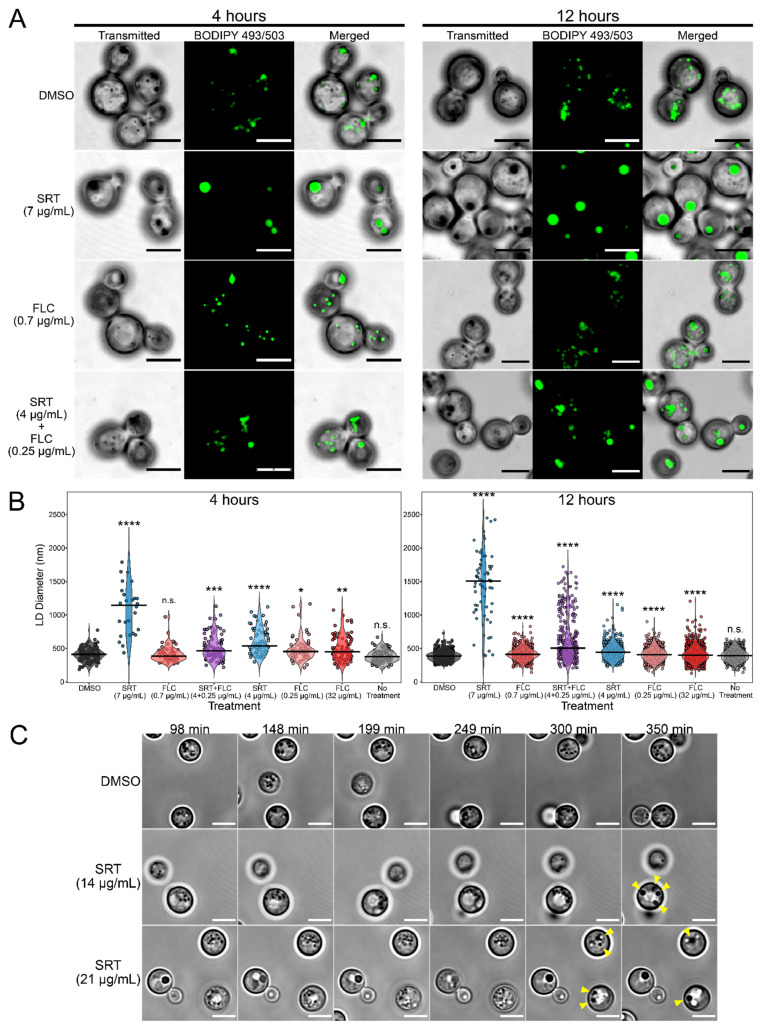
SRT treatment induces the formation of SLDs in *C. neoformans*. (**A**) BODIPY 493/503 staining of SRT- and FLC-treated cells. H99α cells were grown for 12 h in RPMI-1640 at 37 °C with 150 RPM shaking, then resuspended to a density of 5.0 × 10^5^ cells/mL in fresh RPMI-1640 containing the drug. Cultures were incubated for either 4 or 12 h at 37 °C with 150 RPM shaking. At the denoted time points, cells were harvested by centrifugation, fixed with 4% paraformaldehyde, and stored overnight in 1X PBS. Cells were stained with 5 μM BODIPY 493/503 (colored green), loaded onto poly-d-lysine-coated coverslips and imaged. Images shown are projected from Z-stacks; transmitted images were Z-projected by minimum intensity, while fluorescence images are Z-projected by maximum intensity. Scale bars = 5 μm. (**B**) Measurement of LD diameter in cells under various treatment conditions after 4 and 12 h incubation. Black bars denote the median diameter for each treatment. For each time point, significant differences in LD diameter between DMSO and the other treatments were evaluated using pairwise Welch’s *t*-tests. Reported *p*-values were adjusted using Holms’ correction to account for multiple comparisons. * *p* < 0.05; ** *p* < 0.01; *** *p* < 0.001; **** *p* < 0.0001; n.s. not significant. (**C**) SRT-induced SLDs form by the fusion of smaller LDs. LK62 cells were grown overnight in RPMI-1640 and resuspended to 5.0 × 10^6^ cells/mL in fresh RPMI-1640 containing the drug. Ten microliters of cells was loaded onto a poly-d-lysine-coated coverslip within a Hoch chamber. Samples were imaged once every minute. Timestamps at the top of the image denote the time after the drug had been added. Yellow arrows indicate fused structures relative to the previous time point. Scale bars = 5 μm.

We established the concentrations of SRT, FLC, and SRT + FLC needed to reduce *C. neoformans* colony forming units to approximately 50% (Figure S1) after 12 h incubation with these compounds in RPMI-1640-based medium (henceforth RPMI-1640; see Section 2). We chose 7 μg/mL SRT, 0.7 μg/mL FLC, and 4 μg/mL SRT + 0.25 μg/mL FLC as the minimum concentrations in our subsequent experiments. We observed that treatment of *C. neoformans* with 7 μg/mL SRT for 4 h or 12 h resulted in the formation of large and dark spherical intracellular structures that are visible by transmitted light microscopy (Figure 1A). Cells treated with DMSO (vehicle control) or FLC alone closely resembled untreated cells in that only small dark spherical structures were visible (Figure 1A and Figure S2). We hypothesized that the small dark structures were LDs and the large structures were SLDs. To test this, we stained cells with the LD-specific fluorescent dye BODIPY 493/503 and examined the samples by fluorescence microscopy (Figure 1A and Figure S2) [31]. We observed that the BODIPY 493/503 signal localized to the large, SRT-induced structures and the smaller, normal structures, indicating the structures to be SLDs and LDs, respectively. To confirm these results, we stained cells with an additional LD-specific dye, monodansylpentane (MDH), and examined them by fluorescence microscopy [32] (Figure S3). In agreement with the BODIPY 493/503 results, we observed that MDH also localized with structures identified as LDs and SLDs. Thus, we conclude that SRT treatment of *C. neoformans* results in the formation of SLDs.

**Figure 2:**
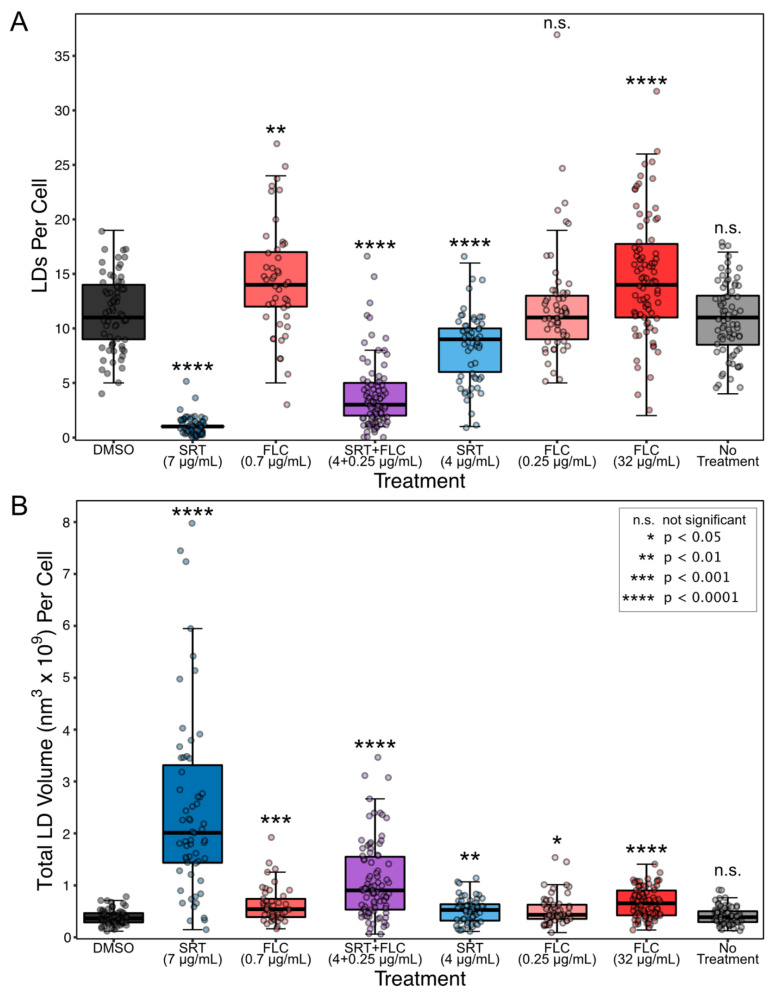
The different drug treatments exert distinct effects on *C. neoformans* LDs after 12 h. (**A**) Effect of SRT on the number of LDs per cell. Significant differences in the number of LDs per cell between DMSO and the other treatments were evaluated using pairwise Welch’s *t*-tests. Reported p-values were adjusted using Holms’ correction to account for multiple comparisons. (**B**) Effect of SRT on the total volume of cellular LDs in *C. neoformans*. Significant differences in the LD volume per cell between DMSO and the other treatments were evaluated using pairwise Welch’s *t*-tests as in (**A**).

To quantitatively assess the effects of SRT on *C. neoformans* LDs, we measured the number of LDs per cell and their diameters following treatment (Figure 1B and Figure 2A). This analysis was consistent with the following observations. First, SLDs in cells treated with 7 μg/mL SRT were generally larger at 12 h than at 4 h, indicating an increase in LD size as a function of time in SRT. Second, co-treatment of cells with 4 μg/mL SRT + 0.25 μg/mL FLC also produced enlarged LDs in some cells, particularly at 12 h (Figure 1A,B). After 12 h, the enlarged LDs from co-treated cells were not as large as those seen in cells treated with 7 μg/mL SRT alone, but these two treatments resulted in the largest observed summed LD volumes in cells (Figure 2B). Furthermore, the LDs in cells co-treated with SRT + FLC were fewer in number and larger than those in cells treated with 4 μg/mL SRT only or with any concentration of FLC alone (Figure 1B and Figure 2A). FLC monotreatment, even at concentrations as high as 32 μg/mL, resulted in only a modest increase in LD size (Figure 1B). Interestingly, treatment with 0.7 or 32 μg/mL FLC increased the number of LDs in cells (Figure 2A) and the summed volume of cellular LDs (Figure 2B). LDs in cells treated with DMSO vehicle alone did not differ from untreated cells by these metrics. Thus, the SLD phenotype observed in cells treated with low concentrations of SRT and FLC appears to be a result of the synergistic effects of the two drugs, such that the SLD phenotype observed with higher SRT is potentiated by FLC with lower SRT. Notably, the synergistic effect of SRT and FLC on SLD formation is consistent with their synergistic anti-cryptococcal effects [11].

The quantification of LDs in BODIPY493/503-stained cells indicated that the formation of SLDs in response to SRT is accompanied by a reduced number or complete loss of normal LDs (Figure 1A and Figure 2A). From these observations, we reasoned that LDs might coalesce to form SLDs in the presence of SRT. We examined SRT-treated cells by time-lapse microscopy under transmitted light (Figure 1C, Videos S1–S3). We observed that, in cells treated with SRT, normal LDs fuse to form larger LDs and that the repeated fusion of increasingly larger LDs leads to the formation of SLDs. While the formation of SLDs in the presence of SRT indicates an impact of SRT on lipid metabolism, SRT did not appear to interfere with the structures of buds once they are formed during cell division, based on the unaltered localization of the bud-site marker CDC10 in budding cells using an mCherry fusion protein (Figure S4).

**Figure 3:**
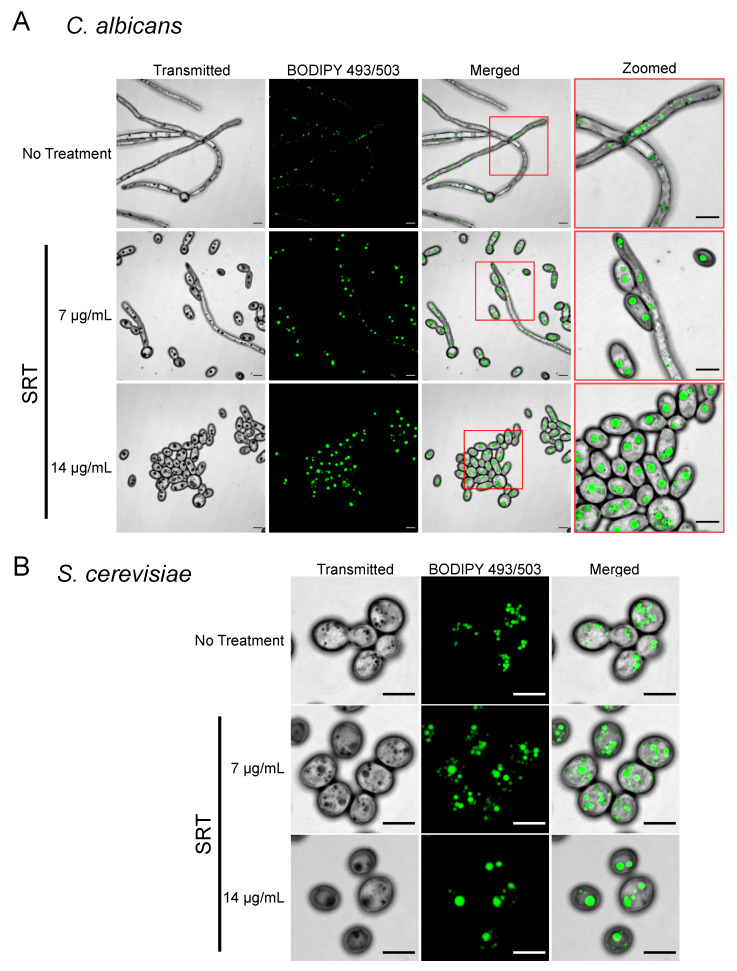
SRT treatment induces SLDs in *C. albicans* and *S. cerevisiae*. (**A**) Treatment with SRT induces the formation of enlarged LDs and reduces hyphal growth in *C. albicans*. SC5314 cells were grown for 12 h in YPD at 30 °C with 150 RPM shaking, then resuspended to a density of 5.0 × 10^6^ cells/mL in fresh RPMI-1640 containing the drug. Treated cultures were incubated for 12 h at 37 °C with 150 RPM shaking. Cells were then harvested by centrifugation, fixed with 4% paraformaldehyde, stained with 5 μM BODIPY 493/503, loaded onto a poly-d-lysine-coated coverslip, and imaged. Images shown are projected from Z-stacks; transmitted images were Z-projected by minimum intensity, while fluorescence images are Z-projected by maximum intensity. Scale bars = 5 μm. (**B**) Treatment with SRT induces the formation of enlarged LDs in *S. cerevisiae*. S288c cells were grown for 12 h in RPMI-1640 at 30 °C with 150 RPM shaking, then resuspended to a density of 5.0 × 10^6^ cells/mL in fresh RPMI-1640 containing the drug. Cultures were incubated for an additional 12 h at 30 °C with 150 RPM shaking. Cells were then harvested by centrifugation, fixed with 4% paraformaldehyde, stained with 5 μM BODIPY 493/503, loaded onto a poly-d-lysine-coated coverslip, and imaged. Images shown are projected from Z-stacks; transmitted images were Z-projected by minimum intensity, while fluorescence images are Z-projected by maximum intensity. Scale bars = 5 μm.

We next asked whether SRT-induced SLD formation is unique to the basidiomycete *Cryptococcus* or is instead representative of a general fungal response to SRT. We again used fluorescence microscopy to examine the lipid responses to SRT, this time in the ascomycetous yeasts *Candida albicans* (Figure 3A) grown at 37 °C and *Saccharomyces cerevisiae* (Figure 3B) grown at 30 °C in RPMI-1640 medium after 12 h. Culturing *C. albicans* in RPMI-1640 media at 37 °C induces filamentous growth [33]. BODIPY 493/503 staining revealed that both *C. albicans* and *S. cerevisiae* cells formed SLDs in the presence of SRT. Strikingly, SRT also substantially reduced the filamentation of *C. albicans*, even at the relatively low dose of 7 μg/mL (MIC_90_ for the strain SC5314 is 32 μg/mL [11]). Finally, we assessed the change in LDs of *Aspergillus fumigatus* conidia in the presence of SRT. We germinated *A. fumigatus* conidia for 0, 8, and 24 h in RPMI-1640 containing SRT. While LDs were detected in conidia prior to treatment, conidia treated with SRT contained a single large LD with a size similar to the size of LDs in hyphae that formed in cultures lacking SRT. Additionally, we noted that SRT strongly inhibited germ tube formation of *A. fumigatus* conidia (Figure S5). Thus, the failure of the conidium to germinate is accompanied by an SLD phenotype in *A. fumigatus*. These results demonstrate that SRT induces SLDs in three additional fungal species, including two major opportunistic pathogens.

## 4. Discussion

The results of this study revealed that treatment with SRT caused the formation of SLDs in *C. neoformans* and that these SLDs formed by the coalescence of smaller LDs. Furthermore, we observed that SRT induced the formation of SLDs in the yeasts *S. cerevisiae* and *C. albicans* and in the filamentous fungus *A. fumigatus*.

One hypothesis to explain our observations is that SRT alters lipid metabolism in *C. neoformans*, such that an increase in the frequency of LD fusion events occurs. A screen of *S. cerevisiae* deletion mutants identified strains that form SLDs constitutively by the fusion of smaller LDs [34]. Nearly all the genes that are affected in these SLD-forming strains function directly or indirectly in phospholipid metabolism [34]. The composition of the LD phospholipid monolayer is an important contributor to LD physiology [28,35]. Importantly, different phospholipids have distinct physical properties which affect the ability of the LD to interact with LDs and other organelles [34,36]. Increasing levels of phosphatidic acid (PA) or phosphatidylethanolamine (PE) are sufficient to induce LD fusion events. In contrast, increasing the intracellular levels of phosphatidylcholine (PC) reduces LD fusion events [34]. Thus, PA and PE are considered to possess fusogenic properties, while PC is thought to exert surfactant properties. Importantly, constitutive SLD-forming *S. cerevisiae* strains exhibit increased amounts of intracellular PA. Therefore, it was proposed that the formation of SLDs in these strains arises from a surplus of PA, which, when integrated into LD monolayers in excess, causes an increase in the frequency of LD fusion events, resulting in the formation of SLDs. Hypothetically, SRT might cause a similar imbalance of phospholipids, resulting in SLD formation. Analyses of SRT’s effects in *S. cerevisiae* grown under different conditions [27] indicated that LDs appreciably increased in number but not in size, which is similar to our observations of the effects of FLC on *C. neoformans* LDs (Figure 1B and Figure 2A). Presumably, global changes in lipid metabolism would also affect membrane-bound organelles, including the endoplasmic reticulum, where LDs form. This remains to be determined.

An alternative explanation for SRT-induced SLD formation is that SLDs form to sequester SRT to protect the cell. SLDs serve as sequestration sites for lipophilic toxins in several fungal species. The endolithic fungus *Phaeosphaeria* sp. synthesizes perylenequinones (PQs) which, upon photoactivation, generate reactive oxygen species that promote pathogenesis by damaging host cell membranes [37]. As a self-protective mechanism, the toxin-producing fungal cells sequester the self-produced PQs to enlarged LDs. Moreover, *Candida* and *Saccharomyces* cells that survive PQ exposure contain SLDs [37]. Induction of SLDs prior to PQ exposure promotes cell survival and, conversely, the absence of LDs increases PQ sensitivity [37]. Similarly, enlarged LDs sequester lipophilic aflatoxin B_1_ in *Aspergillus flavus*, offering self-protection from this mycotoxin [38]. Importantly, SRT is a lipophilic molecule [39]. Thus, it is possible that fungal cells sequester SRT within SLDs.

In this work, we show that treatment with SRT results in the formation of SLDs in *C. neoformans*. Moreover, SRT’s effects on LDs are mimicked in *S. cerevisiae*, *C. albicans*, and *A. fumigatus*, suggesting a conserved effect of SRT on these fungi. While treatment with FLC alone exerts only a modest effect on LD size in *C. neoformans*, a low level of FLC appeared to potentiate SLD formation when combined with a low level of SRT. This effect could reflect the synergistic action that these drugs exhibit to inhibit *C. neoformans* growth [11]. The finding that SRT induces changes in fungal LDs may provide clues to an underlying mechanism by which SRT inhibits fungal growth through its impact on lipid metabolism. In this regard it is important to note that, recently, derivatives of SRT that have more potent anti-cryptococcal activity have been described, and whether they have similar mechanisms of action as SRT itself needs to be determined [40]. Alternatively, SLDs may form to sequester SRT and protect the cell but, in the presence of excess SRT, are ultimately insufficient to protect against its toxicity. At this point it is not clear whether the induction of SLDs is a direct consequence of the antifungal action of SRT. In other contexts, SLD formation is tolerated; constitutive *S. cerevisiae* SLD-formers are viable [34,41] and longer-term growth of *C. neoformans* in oleate, as in other systems, induces SLDs [42]. Finally, it is not yet known whether SLD accumulation is due to metabolic changes generated by SRT or occurs because the drug itself impairs lipid metabolism. In summary, while the causes underlying the formation of SRT-induced SLDs remain unclear, we are not aware of existing antifungal therapeutics that induce SLD formation in fungi, suggesting that SRT exerts novel effects on fungal cells distinct from those of current antifungal agents.

## Data Availability

The data presented in this study are available in this article and in the accompanying Supplementary Material.

## Supplementary Materials

The following supporting information can be downloaded at: https://www.mdpi.com/article/10.3390/jof8060642/s1, Figure S1: Effects of SRT and FLC on *C. neoformans* viability after 12 h treatment; Figure S2: Lower dose SRT treatment induces the formation of SLDs in *C. neoformans* but higher dose FLC treatment does not; Figure S3: MDH staining of SRT-treated *C. neoformans* cells confirms the large dark structures to be SLDs; Figure S4: The formation of SLDs does not alter bud-site localization of CDC10-mCherry in budding *C. neoformans* cells; Figure S5: SRT induces SLD formation in *A. fumigatus* conidia. Video S1: Time-lapse movie of LDs in cells 96–348 min after treatment with DMSO. Video S2: Time-lapse movie of LDs in cells 96–348 min after treatment with 14 μg/mL SRT. Video S3: Time-lapse movie of LDs in cells 96–348 min after treatment with 21 μg/mL SRT. Supplementary File S1: Imaging_chamber.stl defines the geometry of the 3D-printed modified Hoch chamber used for live-cell time-lapse imaging. Supplementary File S1 can be downloaded at DOI:10.6084/m9.figshare.19733851.
