# Supplementary material for: The Antidepressant Sertraline Induces the Formation of Supersized Lipid Droplets in the Human Pathogen Cryptococcus neoformans
Source: J Fungi (Basel). 2022 Jun 17;8(6):642. doi: 10.3390/jof8060642 (PMC9224953; doi:10.3390/jof8060642)
Supplement: Supplementary file 1 [file jof-08-00642-s001.zip › jof-1758308-figures.pdf]

A

B

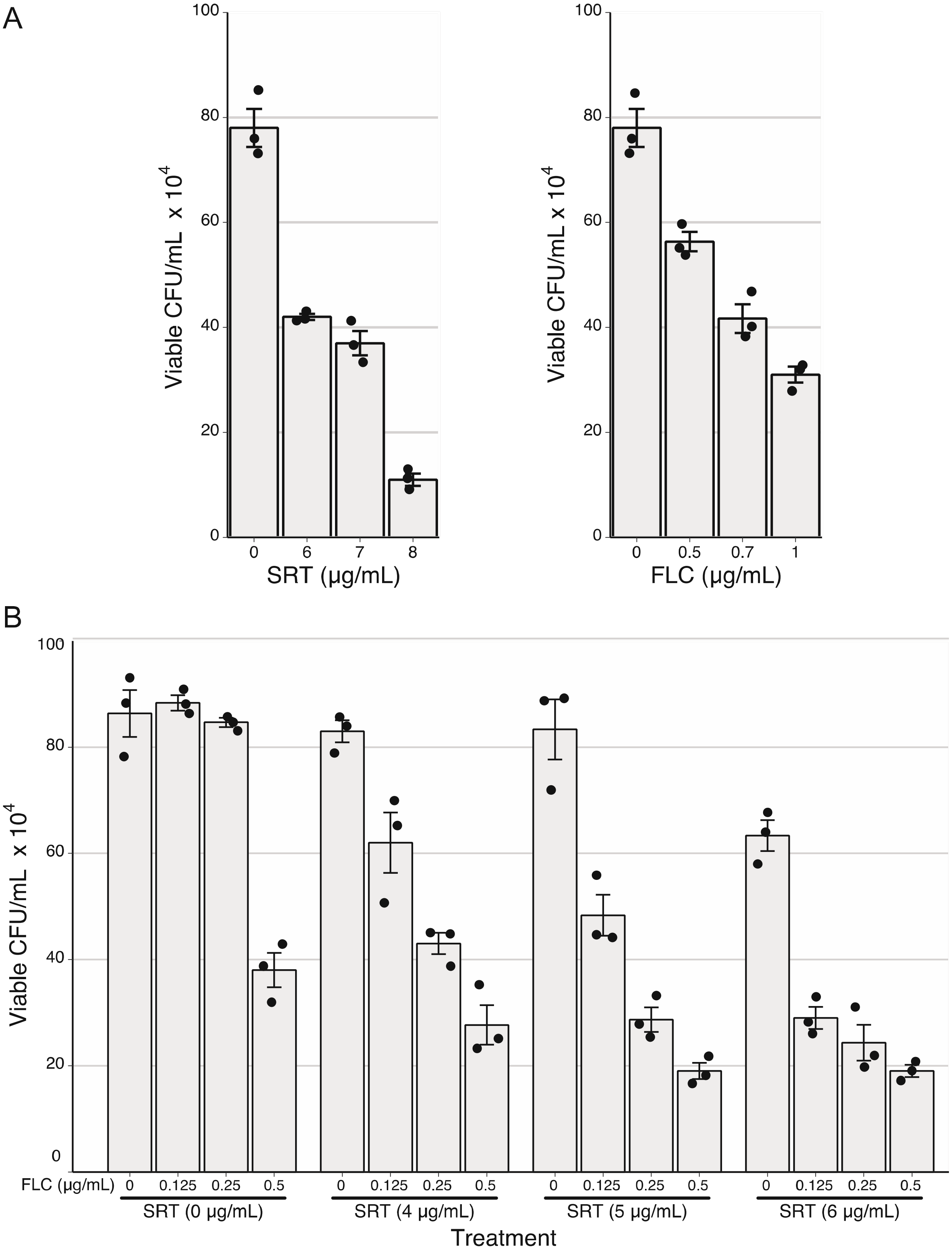

Figure S1. Effects of SRT and FLC on *C. neoformans* viability after 12 hr treatment. Viability assays are described in Materials and Methods. (A) left panel, SRT alone; right panel, FLC alone. (B) Effects of varying SRT and FLC in co-treatment.

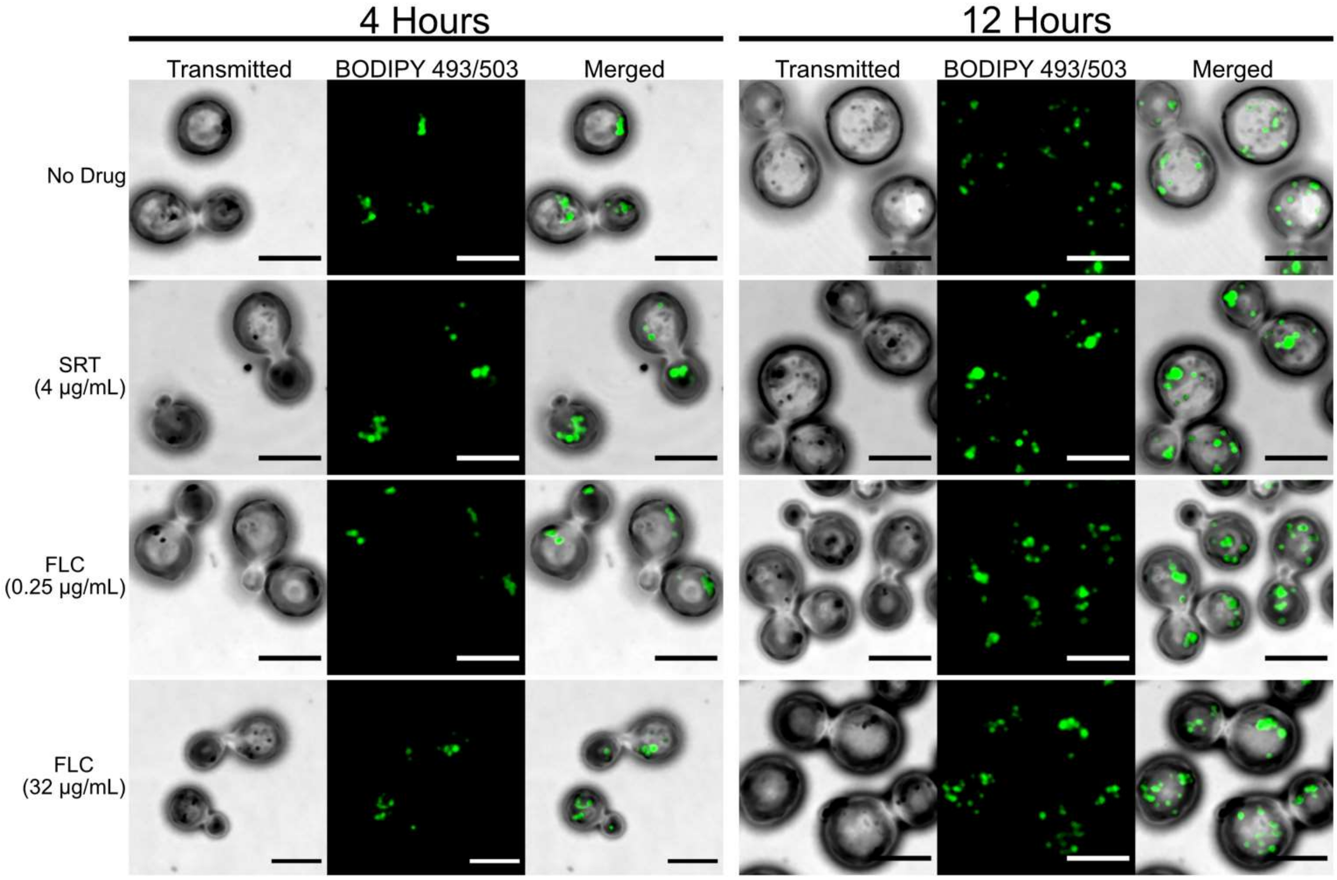

Figure S2. Lower-dose SRT treatment induces the formation of SLDs in *C. neoformans* but higher dose FLC treatment does not. Experimental and imaging conditions are described in Figure 1.

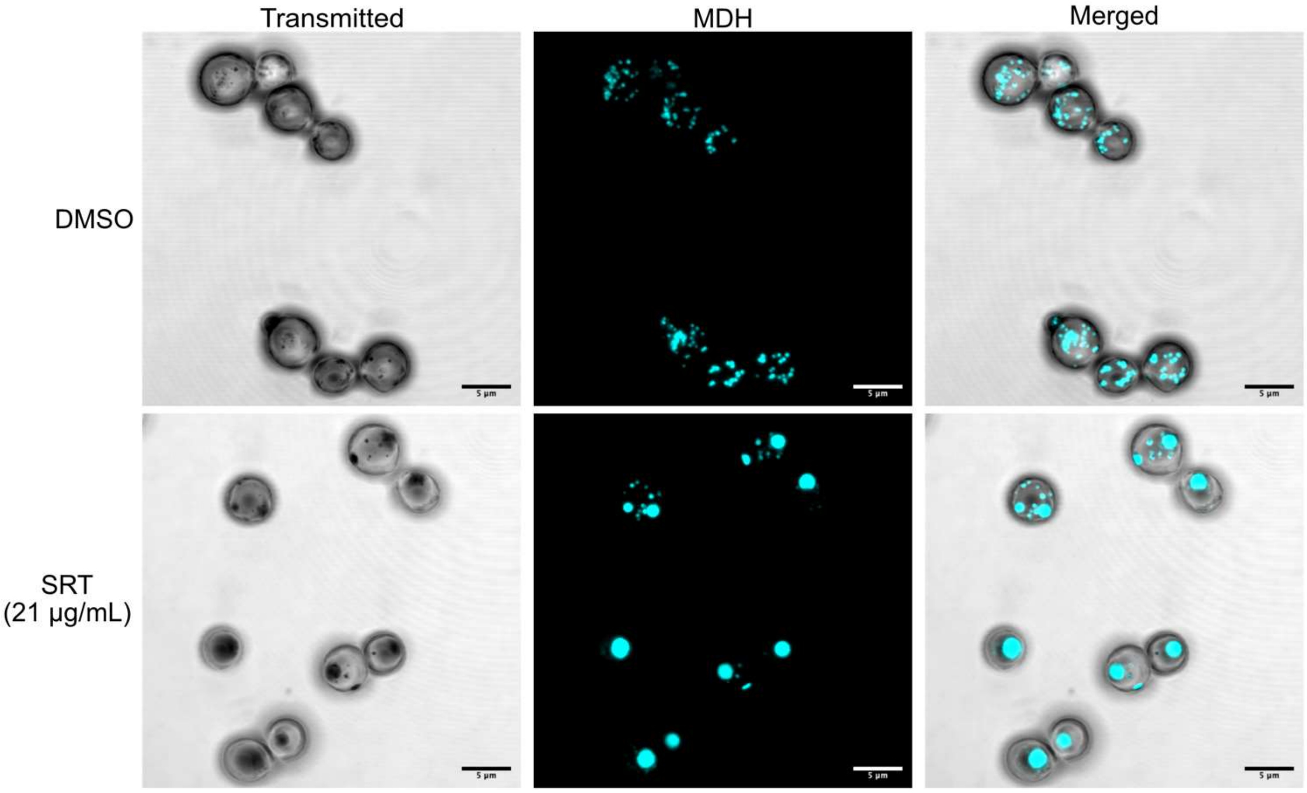

Figure S3. MDH staining of SRT-treated *C. neoformans* cells confirms the large dark structures to be SLDs. H99α cells were grown for 12 hours in RPMI-1640 at 37 °C with 150 RPM shaking, then resuspended to a density of 5.0 x 10^5 cells/mL in fresh RPMI-1640 containing the drug. Cultures were incubated for 14 hours at 37 °C with 150 RPM shaking. Cells were then harvested by centrifugation, stained with 0.1 μM MDH, loaded onto a poly-d-lysine coated coverslip within a Hoch chamber, and imaged. Images shown are projected from Z-stacks; transmitted images are Z-projected by minimum intensity, while fluorescence images are Z-projected by maximum intensity. Scale bars = 5 μm.

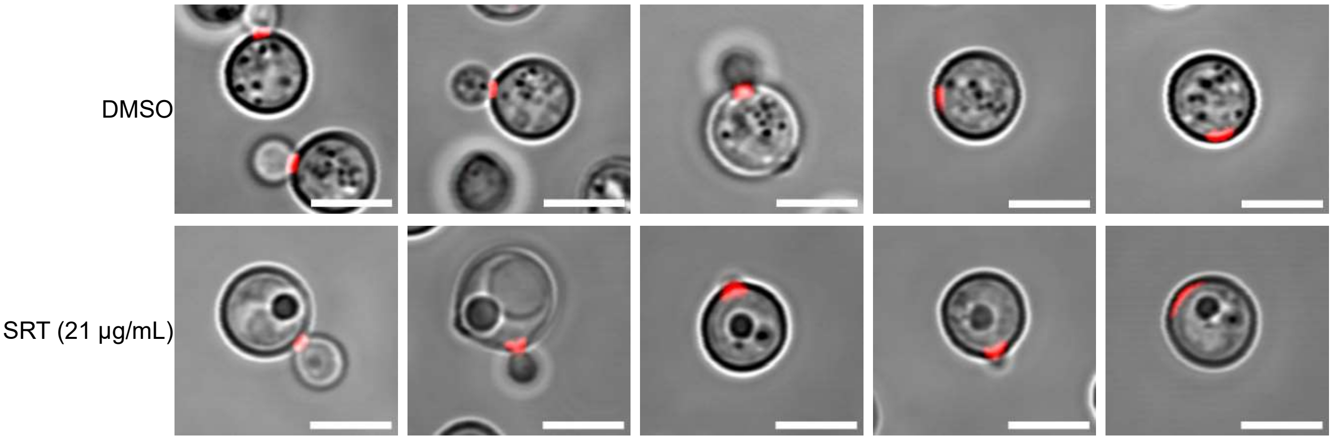

Figure S4. The formation of SLDs does not alter bud-site localization of CDC10-mCherry in budding *C. neoformans* cells. LK62 cells were grown overnight in RPMI-1640 and resuspended to 5.0 x 106 cells/mL in fresh RPMI-1640 containing the drug. Ten microliters of cells were loaded onto a poly-d-lysine coated coverslip within a Hoch chamber and imaged. Each panel represents a different cell. Scale bars = 5 µm.

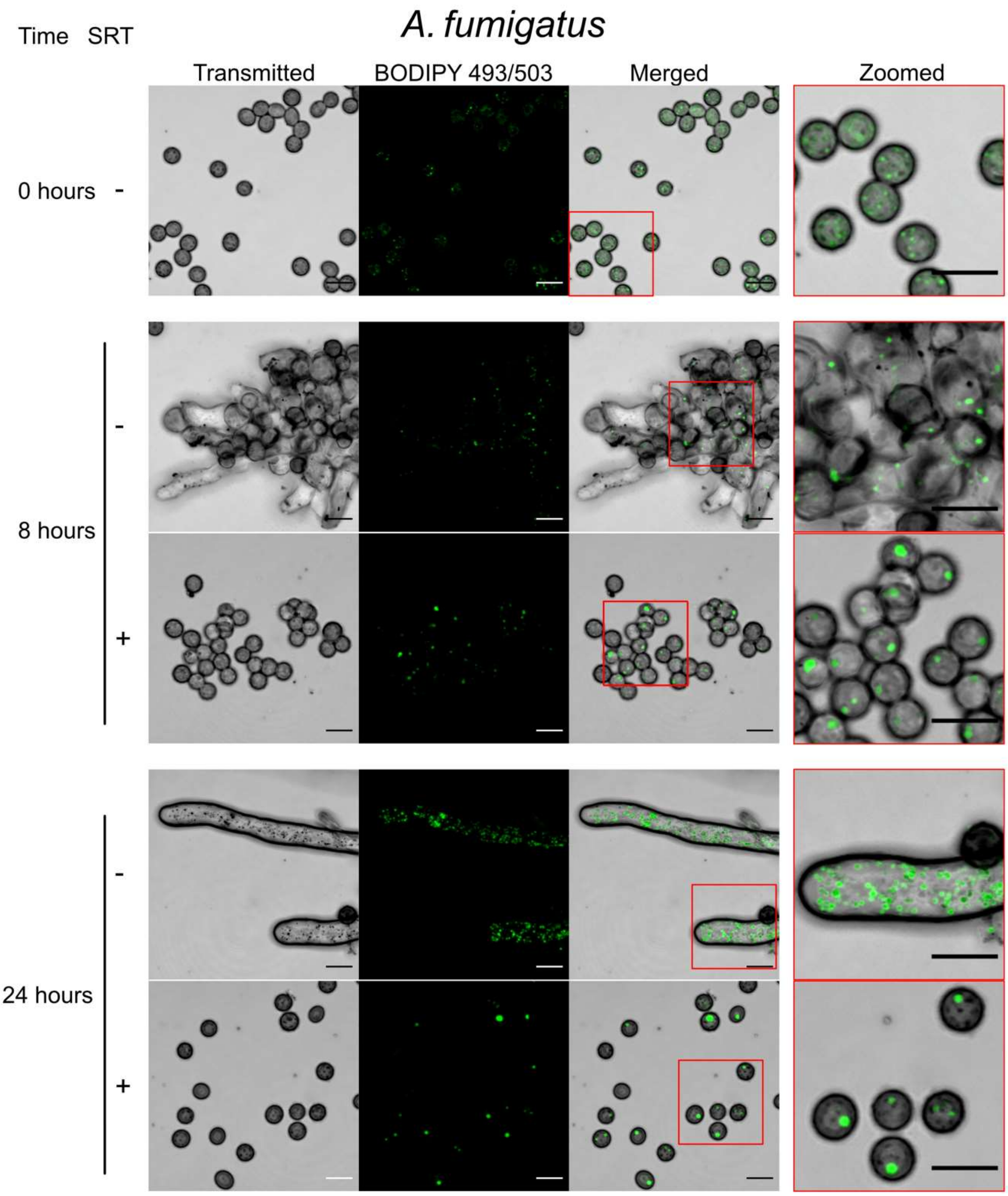

Figure S5. SRT induces SLD formation in *A. fumigatus* conidia. Spores of *A. fumigatus* were obtained from cultures inoculated onto solid Vogel's medium + 1% glucose and incubated for 10 days at 30 °C. Spores were harvested in 1X PBS. The suspension was filtered through several layers of sterile cheesecloth to remove hyphal fragments. Spores were inoculated into RPMI-1640 medium containing either no drug or 28 μg/mL SRT and incubated at 37 °C with 180 RPM shaking for 24 hours. Samples were taken at the indicated time points. Cells were then harvested by centrifugation, fixed with 4% paraformaldehyde, stained with 5 μM BODIPY 493/503, loaded onto a poly-d-lysine coated coverslip, and imaged. Images shown are projected from Z-stacks; transmitted images were Z-projected by minimum intensity, while fluorescence images are Z-projected by maximum intensity. Scale bars = 5 μm.
